# Intervention mapping for development of a participatory return-to-work intervention for temporary agency workers and unemployed workers sick-listed due to musculoskeletal disorders

**DOI:** 10.1186/1471-2458-9-216

**Published:** 2009-07-02

**Authors:** Sylvia J Vermeulen, Johannes R Anema, Antonius JM Schellart, Willem van Mechelen, Allard J van der Beek

**Affiliations:** 1 Department of Public and Occupational Health, EMGO Institute, VU University Medical Center, P.O. Box 7057, 1007 MB, Amsterdam, The Netherlands; 2 Dutch Research Center for Insurance Medicine AMC-UWV-VUmc, VU University Medical Center, Amsterdam, The Netherlands

## Abstract

**Background:**

In the past decade in activities aiming at return-to-work (RTW), there has been a growing awareness to change the focus from sickness and work disability to recovery and work ability. To date, this process in occupational health care (OHC) has mainly been directed towards employees. However, within the working population there are two vulnerable groups: temporary agency workers and unemployed workers, since they have no workplace/employer to return to, when sick-listed. For this group there is a need for tailored RTW strategies and interventions. Therefore, this paper aims to describe the structured and stepwise process of development, implementation and evaluation of a theory- and practise-based participatory RTW program for temporary agency workers and unemployed workers, sick-listed due to musculoskeletal disorders (MSD). This program is based on the already developed and cost-effective RTW program for employees, sick-listed due to low back pain.

**Methods:**

The Intervention Mapping (IM) protocol was used to develop a tailor-made RTW program for temporary agency workers and unemployed workers, sick-listed due to MSD. The Attitude-Social influence-self-Efficacy (ASE) model was used as a theoretical framework for determinants of behaviour regarding RTW of the sick-listed worker and development of the intervention. To ensure participation and facilitate successful adoption and implementation, important stakeholders were involved in all steps of program development and implementation. Results of semi-structured interviews and 'fine-tuning' meetings were used to design the final participatory RTW program.

**Results:**

A structured stepwise RTW program was developed, aimed at making a consensus-based RTW implementation plan. The new program starts with identifying obstacles for RTW, followed by a brainstorm session in which the sick-listed worker and the labour expert of the Social Security Agency (SSA) formulate solutions/possibilities for suitable (therapeutic) work. This process is guided by an independent RTW coordinator to achieve consensus. Based on the resulting RTW implementation plan, to create an actual RTW perspective, a vocational rehabilitation agency is assigned to find a matching (therapeutic) workplace. The cost-effectiveness of this participatory RTW program will be evaluated in a randomised controlled trial.

**Conclusion:**

IM is a promising tool for the development of tailor-made OHC interventions for the vulnerable working population.

## Background

### Participatory interventions and return-to-work

In the past decade in activities aiming at return-to-work (RTW), there has been a growing awareness to change the focus from sickness and work disability to recovery and work ability[1]. In line with this need for a (re)activating approach and the focus on RTW, development of participatory occupational health care (OHC) interventions has received growing attention in recent years [2-7]. To date, studies on the effect of participatory OHC approaches on RTW are limited in number. Participatory approaches in ergonomics as a primary preventive intervention have a longer history and are more established [8-12]. However, when looking at OHC and RTW evidence suggests that participatory ergonomic RTW interventions have a positive impact on: musculoskeletal symptoms, reducing injuries and workers' compensation claims, and a reduction in lost days from work or sickness absence[12]. It is to early to generalize, but the found positive effects on RTW are hopeful [13-15] (Lambeek et al., 2009, submitted). And although the elements of these participatory RTW interventions that contributed most to the favorable outcomes cannot be established based on the above mentioned studies, two key-elements have been suggested[15]. First, the participation of all stakeholders involved in the RTW process, and second stimulating involvement of the sick-listed worker can lead to greater patient control and greater adherence to work modifications.

When looking at the development of participatory RTW interventions, these interventions have to date mainly been directed towards employees[16]. But, within the working population in the Dutch Social Security System there is a vulnerable group: workers who have no workplace/employer to return to when sick-listed.

### The Dutch Social Security System

There are countries where sick-listing can only occur when an individual is gainfully employed. However, in the Netherlands the Sickness Benefits Act provides for workers who are sick-listed and have no (longer) an employment contract. When these workers, i.e. unemployed workers and temporary agency workers, fall ill they can apply for a sickness benefit at the Social Security Agency (SSA) and receive 70% of their last daily wage during the first two years of sickness absence. However, since there is no (longer) a labour agreement, there are no legislative mandates for these workers to be returned to their previous/last job.

Temporary agency work can be considered an atypical and non-standard form of employment. First, there is a triangular relationship (as opposed to the bilateral relationship between an employer and employee) between the worker, a company acting as a temporary work agency, and a user company in which the temporary work agency places the worker at the disposition of the user company. And second, the work is of a temporary nature without a labour agreement, this in contrast to a temporary worker with a fixed-term contract. In the Netherlands temporary workers with a fixed-term contract are viewed as employees and when sick listed the employer has to pay 100% of the daily wage.

### Risk for sickness absence and work disability

Sickness absence and risk for long-term work disability for sick-listed temporary agency workers and sick-listed unemployed workers is higher than for employees [17-19]. One explanation for this is the greater representation of persons with a higher risk for work disability (i.e. lower education, female gender, non-natives and occupationally disabled, i.e. people with developmental or acquired disabilities resulting in occupational impairments) [20-23]. Also, vocational rehabilitation and RTW guidance for this group is unsatisfactory[18,20]. For this group there is a need for tailor-made RTW strategies and interventions (Vermeulen et al., 2009, submitted). However, a participatory RTW program for sick-listed temporary agency workers and sick-listed unemployed workers is not available yet. Therefore, we wanted to develop a participatory intervention for this vulnerable group of workers, sick-listed due to musculoskeletal disorders (MSD). We decided for MSD because this is, next to mental disorders, the second most common cause of work disability among both employees and workers without an employer in the Netherlands[17,24].

### Participatory RTW program for employees with low back pain as starting point

The successful participatory RTW program for employees 2–6 weeks sick-listed due to low back pain[3,15] was the starting point. This program, based on participatory ergonomics (PE)[8,9] consists of a stepwise process to identify and solve obstacles for RTW by the sick-listed employee and his/her supervisor, resulting in a consensus based implementation plan to facilitate RTW. Key element is an independent RTW coordinator who guides the process to achieve consensus. This participatory RTW program resulted in significantly earlier RTW; an average of 27 days. Furthermore, compliance and satisfaction with the intervention were good for employees and OHC professionals. To tailor this RTW program to the needs and specific context of the new target group, i.e. sick-listed temporary agency workers and sick-listed unemployed workers, and to enhance applicability and effectiveness of the program we used Intervention Mapping (IM)[25,26]. This is a six-step iterative process intended to integrate theoretical and empirical knowledge, including input and feedback from multiple stakeholders. To date, IM has been mainly used for health education and health promotion research. Recently, IM has been also applied in the field of OHC and proved to be a promising tool for intervention development[6]. The aim of this paper is to describe the IM process to develop a participatory RTW program for temporary agency workers and unemployed workers, sick-listed due to MSD.

## Methods

**Figure 1:**
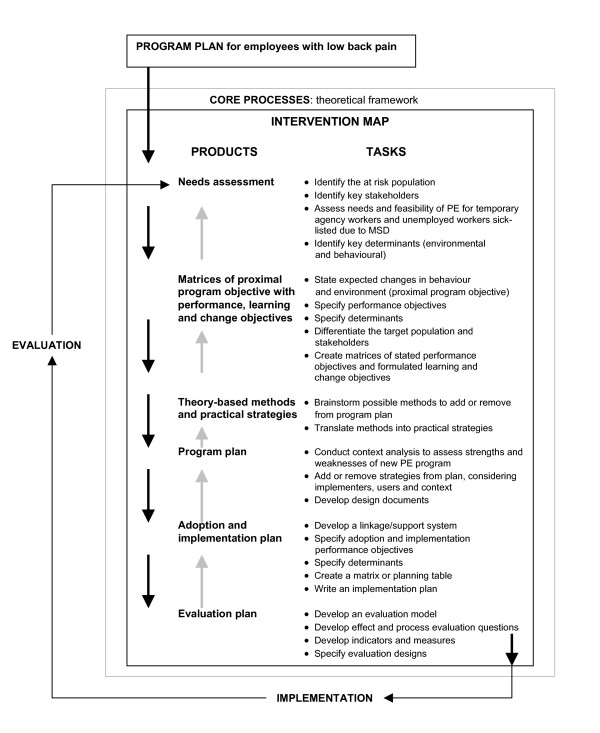
**Intervention Mapping process**. Intervention Mapping process for development of the PE program for temporary agency workers and unemployed workers, sick-listed due to MSD (based on Intervention Mapping as described by Bartholomew and colleagues [25-27].

Intervention Mapping (IM) describes the stepwise process for development of theory- and evidence-based and practise-based interventions [25-28]. The basis for IM is formed by three core processes: searching the literature for empirical findings; assessing and using theory; and collecting and using new data. IM stimulates involvement of stakeholders during the entire process of program development, implementation and evaluation. The Intervention Map itself consists of six steps and, to date, it has been used mainly as a tool for the planning and development of health promotion interventions. IM is an iterative and cumulative process. The program developer moves back and forth between the steps and each step is based on previous steps. In this study, the starting-point was the evidence-based RTW program already developed for employees sick-listed due to low back pain, i.e. the participatory RTW program[3,15]. Next, IM was applied to tailor this participatory RTW program to develop a theory- and practise-based RTW program for a vulnerable group among the working population, i.e. sick-listed temporary agency workers and sick-listed unemployed workers. The six steps of the Intervention Map are described below. In addition, the whole IM process is presented in figure 1.

### Step 1 Needs assessment

The first step in IM is the needs assessment [25-27]. The key purpose of this step was to assess the need for and feasibility of a new RTW program for sick-listed temporary agency workers and sick-listed unemployed workers. The effectiveness of the participatory RTW program has been shown in employees with low back pain [13-15] (Lambeek et al., submitted). However, the target group and involved key stakeholders in this study were significantly different. Therefore, exploration of relevant key stakeholders involved in RTW of sick-listed temporary agency workers and sick-listed unemployed workers in current practise, as well as the needs and feasibility for this type of intervention was conducted. First, the most important stakeholders were the sick-listed temporary agency worker and sick-listed unemployed worker, i.e. the target group. Results from a survey were used to asses the needs among these stakeholders (n = 1077). Next, other important key stakeholders were identified and interviews were held with these stakeholders. They consisted of decision makers from the Social Security Agency (SSA) (n = 3), representatives of the SSA involved in policy regarding the Sickness Benefits Act and Unemployment Insurance Act (n = 5), a decision maker of the Dutch association of temporary work agencies (n = 1), a decision maker of a large temporary work agency (n = 1), and representatives of vocational rehabilitation agencies (n = 3). Based on the needs assessment and a literature review, the new target group (population at risk) and key determinants (environmental and behavioural) for the health problem were identified. Finally, based on this first step, the desired program outcomes were formulated.

### Step 2 Proximal Program Objective

Step 2 of IM is important, because in this step the expected change or program outcome is stated, i.e. who and what will change as a result of the intervention? The main objective of the new program, i.e. the proximal program objective, was defined based upon the needs assessment (step 1) and a scientific analysis of the health problem. Identifying the health problem and associated determinants (environmental and behavioural) in the new target group/population at risk, provided the basis of the new RTW program. Subsequently, performance objectives, learning objectives and change objectives were stated. Finally, matrices were created of these performance objectives, learning objectives and change objectives.

### Step 3 Methods and Strategies

**Figure 2:**
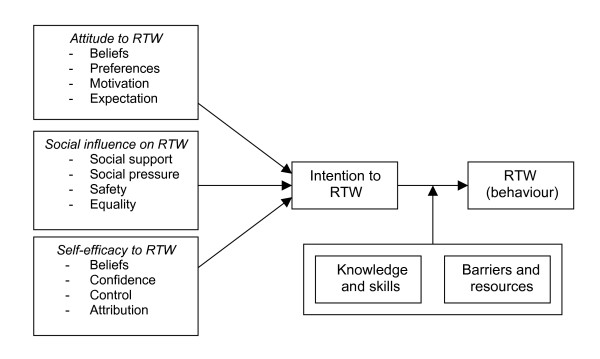
**ASE model applied to RTW of a sick-listed worker**. ASE model regarding RTW of a sick-listed temporary agency worker or a sick-listed unemployed worker, based on the theory of planned behaviour [29]).

The purpose of step 3 of IM is to select suitable theoretical methods and practical strategies to address the learning and change objectives formulated in step 2. Theoretical methods are techniques derived from theory and research, while a strategy is the practical application of a specific method. In selecting methods and strategies several routes may be taken based on experience with theory and practise. Reviewing of the literature showed that RTW of sick-listed temporary agency workers and sick-listed unemployed workers is a rare topic, therefore the general theory approach was used. In line with the development of a participatory RTW program for stress-related mental disorders[6], the Attitude-Social influence-self-Efficacy (ASE) model was chosen as underlying theoretical framework [29-31] for achieving RTW behaviour. This ASE model is based on the theory of planned behaviour[29]. According to this model (see figure 2) the intention regarding RTW behaviour of a sick-listed worker is determined by attitude (views, feelings and preferences of the sick-listed worker regarding RTW), social influence (beliefs, safety, and support of a social network regarding RTW of the sick-listed worker), and self-efficacy (belief of the sick-listed worker that he/she is capable to RTW). In addition, the ASE model includes the influence of barriers and resources, and knowledge and skills to achieve RTW. A review of the literature showed that the three main determinants: worker's attitude, social influence and self-efficacy all have been identified as prognostic factors regarding RTW [32-37].

Next, based on the review of literature, a brainstorm session in the project group, and input from key stakeholder derived from the semi-structured interviews, suitable methods and strategies were chosen. This resulted in a matrix, matching the selected methods and strategies for each determinant.

### Step 4 Program production

In step 4 it is important to verify that the program content matches with the intended target group and program context. To assess the strengths and weaknesses of a participatory RTW program for sick-listed temporary agency workers and sick-listed unemployed workers, a context analysis was conducted[38]. Semi-structured interviews were held with important stakeholders of the SSA, i.e. decision makers (board and management; n = 5), implementers (management and staff; n = 5) and users (insurance physicians and labour experts; n = 17), and representatives of national temporary work agencies (n = 3). Questions were asked regarding the potential benefits of the new RTW program, the complexity of this program, compatibility with daily practise, possibility to try it out, and directly visible results of the new RTW program. Besides analysing the potential of the new program itself, it was also important to take into account the specific factors of the context in which the participatory RTW program will be implemented and used. Therefore, important factors regarding each stakeholder and his/her environment were also analysed, in relation to the individual person (knowledge and skills, self-efficacy, experience, expectations, willingness to change, attitude towards new RTW program, and attitude towards makers of the new RTW program) and the organisation in which they worked (organisation culture, organisation standards and values, organisation structure, degree of policy support, degree of preconditional support, and degree of social and professional support). Each interview was tape-recorded and transcribed. Participants signed a privacy agreement declaring: voluntary participation, no transmittal of information to others, and permission for using this information for the development of the program. The information from these interviews was then used to tailor the participatory RTW program, taking into account the specific target group, the implementers, the users and the specific factors concerning the context in which the program will be applied. Subsequently, two focus group meetings were held to fine-tune the draft version of the new RTW program. These focus groups consisted of representatives of decision makers, implementers and users employed by the SSA. Based on the matrices developed in step 2 and 3, the results of the semi-structured interviews, and the input from the focus groups, a final version of the participatory RTW program for the target group was developed.

### Step 5 Adoption and implementation

Step 5 can be seen as a re-run through the previous IM steps, now focussing on objectives, methods and strategies to ensure the adoption and implementation of the participatory RTW program by the users. Anticipation of implementation is an important factor, ideally starting at the beginning of the IM process. In this step it is required to identify potential users, to formulate adoption and implementation performance objectives for the program users, and to select methods and strategies to achieve the necessary change in behaviour. To achieve successful adoption and implementation in this study, instruction and coaching sessions were held among the users, i.e. OHC professionals. This was supported by purposely developed syllabi with detailed information about the intervention, practical summaries and schemes, and practice material.

### Step 6 Evaluation plan

Step 6 is the anticipation of process and effect evaluation. The list of proximal program objectives, i.e. the main objectives of the new program formulated in step 2, was used as a guidance for the evaluation of the participatory RTW program effects. This resulted in an evaluation plan with defined variables and corresponding evaluation measures.

## Results

### Step 1 Needs assessment

A longitudinal cohort study among sick-listed workers without an employment contract [39-41], constituting of both temporary agency workers and unemployed workers, was used to assess the need of a participatory RTW program for temporary agency workers and unemployed workers, sick-listed due to MSD. Absence of an actual workplace and decreased possibility for RTW in (temporary) adapted work were considered major obstacles and a main reason for the absence of actual RTW [39-41]. Also, satisfaction with OHC by the SSA was moderate[40]. Sick-listed workers without an employment contract reported receiving less OHC interventions than sick-listed employees [39-41]. From their perspective, more could be done by the OHC professionals of the SSA to facilitate RTW. For instance, a problem analysis with making of a RTW implementation plan was viewed as an important OHC intervention. However, only 20% of the sick-listed workers reported receiving this OHC intervention[41]. In contrast to sick-listed employees, there is no legal obligation for employers and temporary agencies regarding RTW support of sick-listed workers without an employment contract. However, among these workers there was a need for structural cooperation regarding RTW with responsibilities for all parties involved, including employers and temporary agencies[41].

**Figure 3:**
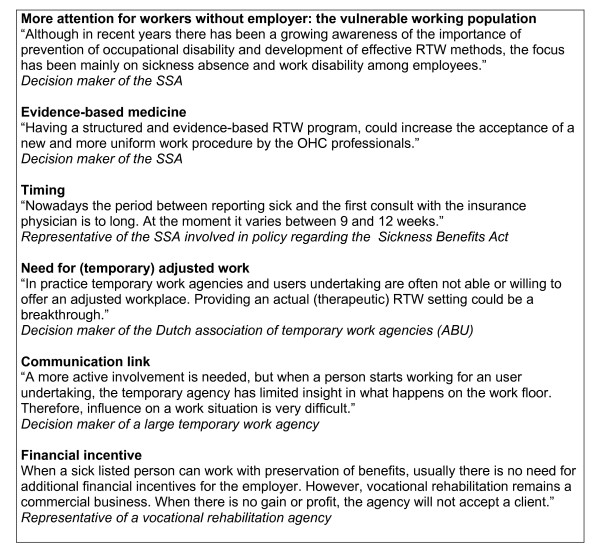
**Illustrating statements derived from the interviews with stakeholders**.

Among the interviewed stakeholders, the need for a new and (cost-)effective RTW program for sick-listed temporary agency workers and sick-listed unemployed workers was commonly shared. Representatives of the SSA involved in policy regarding the Sickness Benefits Act argued that there should be more focus on RTW and on what a disabled worker still can do. Furthermore, decision makers from the SSA emphasized that there is a need for more uniformity and evidence-based interventions. Representatives of the SSA involved in policy regarding the Sickness Benefit Act and Unemployment Insurance Act underlined the need for starting earlier with OHC than current usual care, i.e. between 2 and 4 weeks after reporting sick. In addition, many of the stakeholders viewed also the absence of a workplace to return to a major obstacle for sick-listed temporary agency workers and sick-listed unemployed workers. And although there is a need for (temporary) adjusted work to facilitate RTW for these workers, this is not offered in practice. For the Dutch association of temporary work agencies (ABU) it was important to emphasize "the possibility for temporary work agencies to contribute to their social function and relevance by participating in RTW programs for these sick-listed workers". Since 2003 there is an official covenant between the SSA and the ABU, in which responsibilities for RTW of sick-listed temporary agency workers have been stated. Major themes are attention for the sick-listed temporary agency worker, offering a perspective regarding RTW, and reducing sickness absence. For the decision makers of the SSA and the ABU, minimizing the annual cost of benefit schemes was an important incentive. However, according to the ABU, in daily practice "temporary agency staff are judged on turnover, not on time-consuming rehabilitation support". Moreover, knowledge and experience regarding rehabilitation and RTW of sick-listed temporary agency workers were limited among the temporary agency staff. Structural communication to exchange information, knowledge and experience about OHC and RTW between the SSA and temporary agencies, was viewed as an important and crucial factor in the success of RTW programs for sick-listed temporary agency workers. One of the interviewed vocational rehabilitation agencies had a collaboration with several companies and offered directly available temporary workplaces. The other agencies relied on their network of potential employers, to supply a suitable (temporary) workplace. However, directly available workplaces among the employers in their network were rare. Because searching for a suitable (temporary) workplace and a willing employer takes time, as a result of the interviews it became evident that a financial incentive was needed for the vocational rehabilitation agencies. In figure 3 illustrating statements derived from the interviews with stakeholders are presented.

Summarizing, based on the needs assessment it became clear that the strength of the participatory RTW program was thought to be the consensus procedure to stimulate an active role of the sick-listed worker, to enhance the motivation for RTW and to ensure an adequate match between the temporary work and the capacities/capabilities of the sick listed worker. The possibility of an actual workplace for therapeutic RTW was also viewed as an important key element. Taking into account appropriate incentives for all the stakeholders involved, it was believed to provide an important contribution in RTW of this vulnerable group of workers.

### Step 2 Proximal Program Objective

#### Proximal program objective

Based on the needs assessment and a literature review the proximal program objective, i.e. the main objective of the new program, was formulated: reducing long-term sick-leave and occupational disability for temporary agency workers and unemployed workers, sick-listed due to MSD. Temporary agency workers and unemployed workers with MSD should RTW early and safely by reducing obstacles for RTW and by matching of personal capacities with work(place) demands. Obstacles for RTW can be related to the workplace, work organisation, working conditions, social relations, work environment (mental and/or physical workload), and personal abilities. In the absence of a workplace to return to, a matching temporary (therapeutic) workplace has to be created.

#### Target group and stakeholders

Important stakeholders for a participatory RTW program for sick-listed workers without an employer appeared to be: the temporary agency worker or unemployed worker himself/herself, the OHC providers, i.e. the insurance physician and the labour expert from the SSA as well as the case-manager from the vocational rehabilitation agency or temporary agency. And finally, an important stakeholder in the new participatory RTW program was found to be the RTW coordinator[42], who is an independent person who guides the process towards a consensus-based RTW implementation plan. Involvement of all stakeholders was found to be important, because they all play a key role in the success of RTW of this vulnerable group of workers.

#### Performance objectives

**Figure 4:**
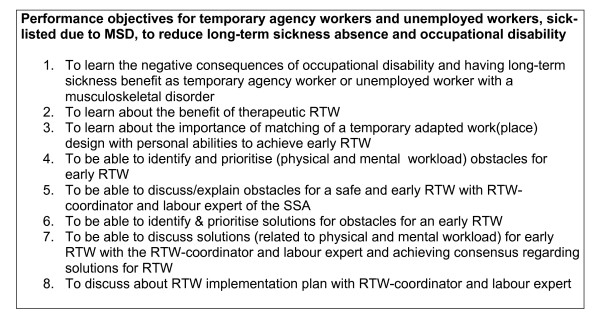
**Performance objectives**. Performance objectives for temporary agency workers and unemployed workers, sick-listed due to MSD, to reduce long-term sickness absence and occupational disability.

The selected performance objectives to reduce long-term sickness absence and occupational disability among temporary agency workers and unemployed workers sick-listed due to MSD are presented in figure 4. Eight performance objectives were formulated for the target group, based on the structure of the participatory RTW program developed for employees sick-listed due to low back pain.

#### Determinants of performance objectives

After stating the performance objectives, the ASE model was used as a framework to describe factors influencing a change in behaviour, i.e. achieving (therapeutic) RTW of the temporary agency worker or unemployed worker. The identified determinants for each performance objective were divided into *personal *determinants (risk perception and knowledge, attitude, skills, self-efficacy, assertiveness, and outcome expectations) and *external *determinants (safety and equality, and support).

#### Learning and change objectives

Finally, based on evidence from a literature review and the needs assessment, matrices were created of the stated performance objectives, and the formulated learning and change objectives. Table 1 shows an example of learning objectives, which belong to the performance objective: the temporary agency worker or unemployed worker will discuss the RTW implementation plan with a RTW coordinator and a labour expert. Table 2 presents an example of change objectives, which belong to the performance objective: the temporary agency worker or unemployed worker is able to identify and prioritise (physical and mental workload) obstacles for early RTW.

**Table 1:** Example of learning objectives

**Performance objective for temporary agency worker or unemployed worker**	**Learning objectives**				
	**Attitude**	**Skills**	**Self-efficacy**	**Assertiveness**	**Outcome expectations**

To discuss about RTW implementation plan with RTW coordinator and labour expert	Positive attitude towards the consensus based RTW implementation plan	Participate in discussion with RTW coordinator and labour expert	Confidence in own ability to discuss with RTW coordinator and labour expert	Dare to participate in discussion with RTW coordinator and labour expert	Having appropriate expectations of (therapeutic) RTW
	Own initiative/motivation for (therapeutic) RTW	Making of realizable appointments regarding persons involved and time scheme for RTW	Confidence in own ability to comply with appointments in RTW implementation plan		
	Belief in positive outcome of PE program				

Learning objectives based on the combination of a performance objective and determinants.

**Table 2:** Example of change objectives

**Performance objective for temporary agency worker or unemployed worker**	**Change objectives**	
	**Safety and equality**	**Support**

To be able to identify and prioritise (physical and mental workload) obstacles for early RTW	RTW coordinator provides clearness about PE process and his/her role	RTW coordinator provides tools to identify and prioritise obstacles (work related and personal factors) for early RTW
	RTW coordinator provides clearness about how to identify and prioritise obstacles for RTW	

Change objectives based on the combination of a performance objective and determinants.

### Step 3 Methods and Strategies

Suitable methods and strategies were selected based on a review of the literature, a brainstorm session in the project group, and input from key stakeholders derived from the semi-structured interviews. Next, these methods and strategies were incorporated in the new RTW program. In table 3 the selected methods and strategies are shown for the determinants risk perception and knowledge, skills and self-efficacy.

**Table 3:** Theoretical methods and practical strategies

**Determinant**	**Methods from theory**	**Strategy**	**Tools/materials**
*Risk perception and knowledge*	Passive learning/providing information	Providing written and verbal information	Letter sent to W about research
			IP explains about personal risk of occupational disability and ending in long term sickness benefit scheme
			Researcher explains participatory RTW program in phone call and sends invitation with folder, IP also explains in first consult.
			RC explains participatory RTW process to W and guides the RTW program
	Active processing of information	Evaluating understanding	IP instructs inventory of RTW obstacles to W as home assignment
			Inventory of RTW obstacles in RTW intervention program

*Skills*	Guided practise	Guided practise	W practises explanation of obstacles to LE with RC
			Practise thinking in broad outline during brainstorm session with RC RC provides post-it notes to stimulate thinking of multiple solutions
		Evaluation	RC checks at the end of the brainstorm session with W if the appointments in the RTW implementation plan are realizable

*Self-efficacy*	Positive reinforcement	Providing feedback	SIP and RC focus on personal abilities and capacities of W regarding RTW
		Evaluation	RC performs an evaluation with W by phone

Matrix of selected theoretical methods and practical strategies for the determinants risk perception and knowledge, skills, and self-efficacy, identified for the PE program. W = temporary agency worker or unemployed worker, IP = insurance physician, LE = labour expert, RC = RTW-coordinator

### Step 4 Program production

#### Context analysis

From the interviews with the users, i.e. OHC professionals (insurance physicians and labour experts from the SSA), it became evident that clear information about and adequate training in using the participatory RTW program was considered important. To avoid delay in starting with the program, appointments had to be made to ensure a quick consult with the insurance physician and labour expert. Additionally, avoiding too much paperwork and supplying adequate computerised support to follow the RTW program were mentioned as relevant success factors. Realizing sufficient support by the staff of the SSA and a structural communication link between all participants by appointing case-managers were also seen as crucial elements. Furthermore, work pressure in daily practise was perceived high and the OHC professionals argued that explicit appointments had to be made with management to ensure sufficient time for implementing and using the new RTW program. Another important precondition was the presence of a RTW perspective for the sick-listed temporary agency worker or sick-listed unemployed worker, by offering an actual workplace for (therapeutic) RTW. In addition, the decision makers advised to ensure adequate overall implementation support by appointing a fulltime project manager. And the staff of the SSA emphasized the importance of having an independent person to guide the process towards a consensus based RTW implementation plan. Also, clear appointments about financial rewards for vocational rehabilitation agencies were seen as an important precondition to ensure the presence of RTW perspective for these vulnerable workers. Finally, from the perspective of the temporary work agencies it was important to have a worker who is directly employable. This meant that the RTW implementation plan could be matched with existing vacancies. The results of the semi-structured interviews and input from the 'fine-tuning' meetings with the OHC professionals, staff and management of the SSA were used to design the final participatory RTW program.

#### Processing of program plan

Important elements from the needs assessment that have been incorporated in the RTW program are: the making of a RTW implementation plan with active involvement of the sick-listed worker and matching of possibilities with capacities; creating an actual (therapeutic) workplace; focus on what a disabled worker still can do; starting earlier with OHC; facilitating structural communication between the SSA, the temporary work agency and the vocational rehabilitation agency and; supplying a financial incentive for the vocational rehabilitation agency. In addition, as a result of the context analysis, i.e. the semi-structured interviews, the following items were incorporated: an appointment was made to ensure a quick consult with the insurance physician; an appointment was also made to ensure that the OHC professionals had sufficient time to work with the new RTW program; a specifically tailored computerised support system was developed; case-managers were appointed for structural communication between all parties involved and; a fulltime project manager was appointed.

As a result of the needs assessment, the semi-structured interviews and input from the focus groups, the existing participatory RTW program for employees sick-listed due to low back pain was adapted and resulted in a participatory RTW program for temporary agency workers and unemployed workers sick-listed due to MSD. First, the sick-listed worker is an essential stakeholder. Another important stakeholder in the RTW program for sick-listed employees is the supervisor at the workplace. Since in most cases the sick-listed temporary agency worker or sick-listed unemployed worker has no employer, there is also no formal supervisor. For this group of sick-listed workers, the SSA is responsible to facilitate RTW: the insurance physician has the role of OHC professional and the labour expert has the role of case manager in vocational rehabilitation support. Thus, the labour expert of the SSA is the second important stakeholder in the new RTW program. Finally, a key role in the participatory RTW program was found for the RTW coordinator[42], who guides the process towards a consensus based RTW implementation plan. This person has to have good process guiding abilities, an independent position, and sufficient knowledge and experience regarding rehabilitation. The labour experts of the SSA fulfilled these requirements. To guarantee the independence of the RTW coordinator, it was stated that he/she should have no other involvement in the rehabilitation support of the sick-listed worker concerned. Table 4 shows an overview of the new participatory RTW program. Additional points of interest were found for each step and are described below.

**Table 4:** Structure of the PE program

**Step**		**Content**	**Who are involved?**
*1*.	*Organisation and preparation*	Check if insurance physician and labour expert have been informed about program and agree with it	RTW coordinator
		Check if combined consult with insurance physician and labour expert is planned	RTW coordinator
		Check who is case manager of vocational rehabilitation agency for placement in temporary (therapeutic) work	RTW coordinator
		Plan appointments for conversations	RTW coordinator, worker and labour expert

*2*.	*Inventory of obstacles and experienced limitations regarding RTW*	Interviews about work tasks, obstacles and experienced limitations for RTW	RTW coordinator has separate interviews with worker and labour expert
		Prioritize obstacles and limitations for return-to-work	RTW coordinator, worker and labour expert

*3*.	*Inventory of (therapeutic) work possibilities (thinking of and choosing solutions*	Thinking of and collecting solutions for suitable (therapeutic) work (places)	RTW coordinator, worker and labour expert
		Prioritizing solutions	RTW coordinator, worker and labour expert

*4*.	*Preparation of matching (temporary) work(place) and reporting*	Make plan for implementation of solutions i.e. placement in matching (therapeutic) work	RTW coordinator, worker and labour expert
		Stimulate own initiative of worker. While waiting on placement by agency, worker can also search for a suitable workplace	RTW coordinator, worker and labour expert
		Contact with vocational rehabilitation agency for intake	RTW coordinator, worker and case-manager of vocational rehabilitation agency
		Intake with vocational rehabilitation agency	Case-manager of vocational rehabilitation agency and worker If desired also RTW coordinator

*5*.	*Placement in matching (therapeutic) work and support*	Placement in matching (therapeutic) workplace	Case-manager of vocational rehabilitation agency, worker and employer
		If necessary, information and instruction at new workplace	Case-manager of agency, worker and employer

*6*.	*Evaluation/control*	Evaluation by phone: has placement in matching (therapeutic) work been realised? Satisfaction with placement in (therapeutic) work? Are adjustments necessary?	RTW coordinator has separate evaluations with worker and labour expert
		If placement has not yet been realised: stimulate own initiative of worker to find a suitable work(place)	Case-manager of rehabilitation agency also evaluates separate with worker and provides feedback to RTW coordinator

#### 1. Organisation and preparation

To ensure that the (labour expert in the role of) case-manager in the participatory RTW program has sufficient information regarding the sick-listed worker, the sick-listed worker always has a consult with the labour expert before the start of the program. For practical reasons, and to minimize the inconvenience for the sick-listed worker, this consult directly follows the first consult with the insurance physician.

To stimulate an active involvement of the sick-listed worker in the participatory RTW program, the insurance physician asks to make an inventory of RTW obstacles, whether it be work or non-work related, as a home assignment in the first consult. The sick-listed worker is also asked to indicate to what extent the obstacles can be influenced. This inventory can be used as a starting point in the interview with the RTW coordinator.

#### 2. Inventory of obstacles and experienced limitations regarding RTW

Adequate introduction by the RTW coordinator is important. The RTW coordinator underlines his/her independence, and stresses that guiding the participatory RTW process with equal contribution of the sick-listed worker and the labour expert is his/her main goal.

#### 3. Inventory of (therapeutic) work possibilities (thinking of and choosing solutions)

In the planned brainstorm session the RTW coordinator, the sick-listed worker and the labour expert formulate solutions/possibilities for suitable (therapeutic) work. These solutions/possibilities can include aspects regarding work content, workplace, work organisation, work conditions and/or work environment. Since there is (in most cases) no workplace to return to, an extra element was added to the program. To provide an actual workplace, agreements were made with four vocational rehabilitation agencies. Within four weeks after enlisting, the assigned vocational rehabilitation agency has to offer at least two suitable therapeutic workplaces matching with the RTW implementation plan. If these suitable workplaces are not offered within the four week period, the other vocational rehabilitation agencies are asked also to search for suitable workplaces.

#### 4. Preparation of matching (temporary) work(place) and reporting

As a conclusion of the above mentioned brainstorm session, the RTW coordinator makes a report in which the main items of the participatory RTW process are described: a summary of prioritised obstacles for RTW, the consensus based solutions, and if possible a concrete work(place) profile. In this RTW implementation plan explicit arrangements are formulated, including a concrete time path. Who does what and when? This report is then sent to the sick-listed worker, the labour expert and the insurance physician. And finally, the RTW coordinator informs the case-manager of the assigned vocational rehabilitation agency.

#### 5. Placement in matching (therapeutic) work and support

The vocational rehabilitation agency has the task to find a (therapeutic) workplace, matching with the profile in the RTW implementation plan. A financial reward is given by the SSA to the vocational rehabilitation agency for placement in a matching (therapeutic) workplace.

#### 6. Evaluation/control

The RTW coordinator evaluates approximately six weeks after making the consensus-based RTW implementation plan to see if everything is going according to plan. This is then registered in a final report and send to the sick-listed worker, the labour expert and the insurance physician.

### Step 5 Adoption and implementation

As mentioned above, important stakeholders were involved in development of the new participatory RTW program to facilitate successful adoption and implementation. Next, purposely developed instruction and coaching sessions were held among the users, i.e. OHC professionals. All involved professionals received a syllabus with detailed information about the program, the participatory RTW protocol, practical summaries and schemes, and practice material. An additional training was developed for the RTW coordinators. The coaching for all involved professionals focused on: content of the protocol, role of the insurance physician, role of the labour expert, placement in (therapeutic) work by the vocational rehabilitation agency, and a brief instruction regarding the for this project developed computerised support system. The additional training for RTW coordinators focused on: content of the protocol, role of the RTW coordinator with illustrations for each step, and practise with anonymous cases and reporting. All professionals were offered personal guidance with the first cases to facilitate working with the new RTW program. Also a follow-up session was held with all participating multidisciplinary teams separately, consisting of the RTW coordinator, the labour expert and the insurance physician, to discuss difficulties and problems with working with the new RTW program in practise. A second follow-up session was held with all involved professionals together, including staff and management. This session was aimed at briefly refreshing the content of the participatory RTW program and to practise with cases as the main purpose.

Finally, to ensure adequate overall implementation support a project manager was appointed. Also a team to guide the process of implementation was formed, consisting of the researchers, representatives of the staff and management of the SSA, including the project manager, and representatives of the participating vocational rehabilitation agencies to facilitate adoption and implementation.

### Step 6 Evaluation Plan

The (cost-)effectiveness of the new participatory RTW program will be evaluated in a randomised controlled trial. In addition, the implementation process will be evaluated. The Medical Ethical Committee of the VU University Medical Centre (Amsterdam, the Netherlands) has approved the study protocol. Trial registration: NTR1047. The results will be described elsewhere.

## Discussion

The aim was to describe the development, implementation and evaluation of a theory- and practise-based participatory RTW program for a vulnerable group among the working population, i.e. temporary agency workers and unemployed workers, sick-listed due to MSD. Following each IM step carefully, made it possible to tailor the existing participatory RTW program, taking into account the specific target group, the implementers, the users as well as the context in which the new participatory RTW program will be applied.

### Strengths

IM proved to be a useful tool to map the path from needs and feasibility to a specifically tailored participatory RTW program. Because implementation of evidence-based interventions in OHC has been difficult, there is a need for systematic documentation of intervention development and implementation research[43]. Going back and forth between the IM steps made it possible to carefully consider each decision in the development, implementation and evaluation of the new program. And since the degree to which a project is planned is an important factor for its potential success[44], we believe that following all IM steps will enhance applicability and future implementation. Furthermore, there is a growing need to optimize the role of stakeholders in OHC research, including intervention development and implementation [45-49]. In line with this, the IM protocol strongly supported input from different stakeholders to ensure participation and involvement in all steps of program development and implementation.

Another strength of this study is the use of the ASE model [29-31] as an underlying theoretical framework for determinants of behaviour regarding RTW and development of the intervention. This is strongly supported by recent insights regarding conceptual models for RTW, arguing that there is a need for a commonly adopted paradigm[50,51].

In addition, the new participatory RTW program was specifically tailored for the target group, the users and the context. By discussing with stakeholders e.g. in focus groups about important factors for innovations, such as potential advantage, complexity of the new program and compatibility with daily practise, we believe that this will enhance the success of future implementation[38].

Finally, in our opinion, following a time-consuming intervention development process, i.e. IM, instead of choosing a more haphazard approach to intervention design, led to innovations that otherwise would have been missed. For instance, the development of a specifically computerised support system, and making of explicit appointments with the management to ensure sufficient time for the OHC professionals to work with the new program. We believe that following the IM process resulted in a combination of keystones to be incorporated in the new participatory RTW program, which will enhance the commitment of the stakeholders and the implementation of the intervention by tailoring the intervention to their needs and the specific context.

### Weaknesses

In this study the contribution of the intended target group itself was relatively modest compared to other stakeholders. Because the program has to be carried out by the OHC professionals of the SSA, the majority of involved persons in IM were from the SSA. It is possible, that the IM process would have resulted in other changes of the participatory RTW program if temporary agency workers and unemployed workers, sick-listed due to MSD, would have played a larger role in program development. However, when looking at the results of a longitudinal cohort study among sick-listed workers without an employment contract [39-41], which was used for the needs assessment, the new participatory RTW program contains many of the elements mentioned in this study by the sick-listed temporary agency workers and sick-listed unemployed workers. This new RTW program stimulates early RTW intervention, more contact with the OHC professionals of the SSA, making of a consensus based RTW implementation plan, the presence of a (therapeutic) workplace to RTW, and structural communication between all parties involved. Therefore, we believe that the new RTW program matches the need of this vulnerable group for tailor-made OHC interventions. However, it will be difficult to generalize this RTW program to another context.

### Comparison with other studies

Development of OHC interventions is a relatively rare described topic in the international literature. The few publications[4,52,53] are based on a three phase process: development, implementation and evaluation, as proposed by Goldenhar and colleagues[43]. The importance of participatory strategies in program development has been also underlined by others [54-56]. In contrast to these studies, the main strength of IM for development of OHC interventions is the combination of a theory-based framework, choosing practical strategies and stimulating active involvement of all stakeholders during the whole process of program development, implementation and evaluation [25-27]. To our knowledge this is the first study, which has applied IM for intervention development for a vulnerable working population, consisting of temporary agency workers and unemployed workers.

### Recommendations

To date, IM has been mainly used as a tool for the planning and development of health promotion interventions [25-27]. Recently, promising results were shown for the use of IM in OHC research[6]. This study shows that IM can also be useful for development of intervention programs for vulnerable working populations.

In addition, further development of other occupational disability interventions for the vulnerable working population, i.e. workers without an employment contract, is needed. Since these workers do not have a permanent workplace/employer to return to when they are sick-listed, there is a need for new interventions which focus on RTW possibilities and which provide an actual RTW perspective for this group of workers. IM seems a promising tool to tailor new interventions to the specific needs and context and to enhance applicability and effectiveness of these programs.

## Conclusion

Following all IM steps resulted in a structured stepwise participatory RTW program for temporary agency workers and unemployed workers, sick-listed due to MSD. The implementation process and the cost-effectiveness regarding this new intervention will be evaluated in the near future.

The results will be available in 2010.

## Competing interests

The authors declare that they have no competing interests.

## Authors' contributions

SJV, JHA and AJMS were involved in development of the participatory RTW program. SJV and JHA were responsible for the general coordination of the study and the implementation of the participatory RTW program. SJV drafted the manuscript. All authors have read and corrected draft versions of the manuscript and approved the final manuscript.

## Pre-publication history

The pre-publication history for this paper can be accessed here:


